# Future Projections of Diabetes-Related Amputations in Eastern Saudi Arabia During 2022–2045 Using a Validated Epidemiological Model

**DOI:** 10.7759/cureus.45972

**Published:** 2023-09-26

**Authors:** Abdulkareem J Al-Quwaidhi, Essa M AlSaleh

**Affiliations:** 1 Preventive Medicine, Al-Ahsa Health Cluster, Ministry of Health, Al-Ahsa, SAU; 2 Infection Control, Al-Ahsa Directorate of Health Affairs, Ministry of Health, Hofuf, SAU

**Keywords:** projections, saudi arabia, modeling, diabetes, amputations

## Abstract

Introduction: Diabetes-related amputations (DRA) are associated with significant morbidity and mortality. There are limited studies on the burden of this condition and its future projections in Saudi Arabia.

Objectives: To estimate future forecasts in the burden of DRA (number of cases and mortalities) among adult diabetics (aged ≥20 years) in Al-Ahsa, Eastern Region of Saudi Arabia from 2022 to 2045.

Methods: A simulation epidemiological model was designed and validated. It is a simple discrete-state model composed of multiple states, in which diabetics make annual transitions to either 'Major Amputations', 'Minor Amputations', or 'No Amputations' states, and then to two states of mortalities. The data inputs required are minimal, including the total diagnosed cases of diabetes for 2022 and transition parameters obtained from recent published literature. The model used some reasonable assumptions and scenarios for testing potential uncertainties around the model outputs. Model validation was conducted by comparing its estimates with the observed local data from two main hospitals in Al-Ahsa for 2022.

Results: The model projected that the total number of DRA among diabetics in Al-Ahsa will increase from 129 (uncertainty interval (UI): 103-154) in 2022 to 169 (UI: 136-203) in 2030 and 227 (UI: 182-272) in 2045, assuming that the incidence rates of major and minor amputations among diabetics will remain constant. However, assuming that these incidence rates will show a gradual decline of 20% every three years, the model predicted the total number of DRA to decrease from 103 (UI: 82-124) in 2022 to 91 (UI: 73-110) in 2030 and 61 (UI: 49-74) in 2045.

Conclusion: DRA impose a considerable burden on patients and the healthcare system, despite the possibility of a potential decrease in incidence rates.

## Introduction

It is currently clear that diabetes is a major public health issue in the Middle East and North Africa (MENA) region, including the Kingdom of Saudi Arabia (KSA). According to the International Diabetes Federation (IDF), approximately 4.3 million people aged 20-79 years in KSA had diabetes in 2021, and this number is expected to increase to 7.5 million by 2045 [1]. This remarkable burden is mainly attributed to accelerated urbanization and changes in the population's lifestyle toward sedentary behaviors and unhealthy dietary patterns [2].

Diabetes is associated with multiple complications that can lead to death or affect the quality of life of patients. Diabetes-related amputations (DRA) are indeed one of these serious complications and are linked to tremendous morbidity and mortality [3]. IDF estimated that more than one in five people are affected with diabetes and foot ulcers or amputations in Africa and the Middle East, comprising the highest number among all other world regions [4]. IDF also reported that the prevalence of recurrence of diabetic foot ulcers in the MENA region ranged between 31.0% in Egypt and 43.0% in Turkey. Moreover, the prevalence of lower limb amputations in the MENA region varied from as low as 0.2% in KSA to as high as 60.0% in Jordan. These wide variations were explained by different sampling methods used, where some studies appeared to have recruited patients from specialty clinics or inpatient high-risk settings, rather than community settings [4]. In a recent systematic review and meta-analysis, it has been estimated that the overall pooled amputation prevalence rates among individuals with diabetes and with diabetic foot ulcers in the Middle East were 2% and 33%, respectively [5].

In KSA, studies on the burden and magnitude of DRA are generally scarce and patchy. One study estimated that the national prevalence of amputations was 1.06% among a sample of 62,681 diabetic patients aged ≥25 years in 2015 [6]. Most of the other studies were conducted inside certain hospitals in Riyadh and Jeddah among patients with diabetic foot ulcers, and some of them recruited only small sample sizes. Therefore, there are large variations in the reported prevalence rates of DRA in these studies. For example, in Jeddah, prevalence rates of DRA among the sampled diabetic foot patients were 60% in 2008 [7], 29.7% in 2015 [8], and 84.9% during 2013-2020 [9]. In Riyadh, a study conducted in a tertiary rehabilitation center between 2010 and 2020 estimated that there were 1,409 amputees, with diabetics constituting more than 40% of them [10].

In Eastern Saudi Arabia, one of the highly urbanized regions, studies related to DRA are limited. One study reported a prevalence rate of DRA at 19% among only 62 patients with diabetic foot ulcers at Saudi Aramco Medical Services Organization in 2004 [11]. Another study was carried out among 82 patients at the Armed Forces Hospital in Dhahran in 2015-2016 and estimated a DRA prevalence rate of 40.2% [12].

Modeling studies have been extensively used in the fields of epidemiology and public health [13]. They are used mainly to guide policy decisions in many areas that affect human life and health [13]. Epidemiological models can be used to predict the trends in the prevalence and mortality of a health condition under alternative scenarios. This makes models a good platform to examine and compare various future policy options and scenarios within a population for appropriate decision-making, planning, and resource allocation [14]. One of the advantages of modeling is its ability to use different types of inputs from various sources and to reveal the logical connection between these inputs and outputs of interest. A model can accommodate data from prevalence studies, prospective studies, controlled trials, meta-analyses, routine surveillance, expert opinions, and assumptions [15]. However, different types of data sources are most likely associated with uncertainty of the model outputs. Therefore, models must be subjected to sensitivity analyses to identify the impact of potential uncertainties around the different input parameters [16].

In this study, an epidemiological model was designed and validated to predict the future trends in the DRA in Al-Ahsa, Eastern Saudi Arabia till 2045. The model integrated various data inputs from the relevant local health departments, published evidence, and assumptions. It was exposed to sensitivity analyses and validation processes to test its ability to predict the future burden of DRA in Al-Ahsa.

To the best of our knowledge, this is the first study in KSA that attempted to use a modeling approach in projecting the trends in DRA over the next two decades. The model in this study can form a starting point in measuring the problem burden in other regions of KSA and even across the national level.

## Materials and methods

Model structure

**Figure 1:**
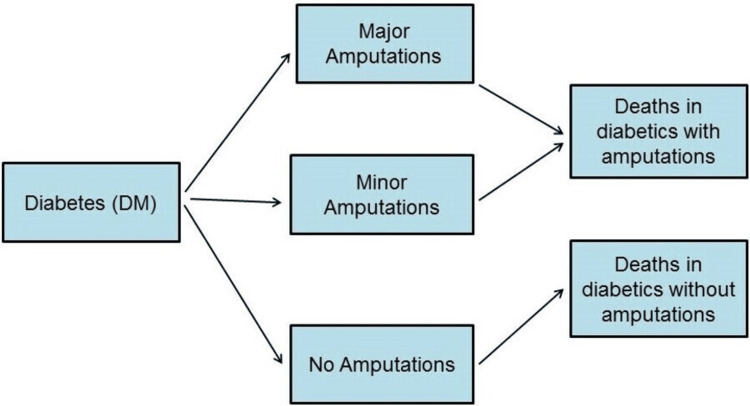
Structure of the model

A simulation epidemiological model was designed for this study to estimate the future forecasts for the burden of DRA (number of cases and mortalities) in Al-Ahsa over the period from 2022 to 2045. It is a simple discrete-state model implemented in Microsoft Excel spreadsheets. It is composed of multiple compartments or states, in which individuals with diabetes make annual transitions to either 'Major Amputations', 'Minor Amputations', or 'No Amputations' states, and then to two different states of mortalities (Figure 1). The model requires a minimal amount of data inputs, which include the total number of diagnosed diabetes cases aged ≥20 years in Al-Ahsa for 2022 (obtained from the Community Wellness Department, Al-Ahsa Health Cluster), in addition to some few epidemiological parameters to inform the transitions of individuals between the model states. These parameters were the annual incidence rates of major (above ankle) and minor (below ankle) amputations among individuals with diabetes, in addition to the all-cause mortality rates among amputees and non-amputees. Transition parameters were obtained from the literature after a comprehensive review, with priority given to national estimates if available (as the case with all-cause mortality rates) [17], or using recent pooled global estimates from a systematic review and meta-analysis if national data are lacking (as the case with incidence rates of amputations among diabetics) [18]. In addition to transition parameters, the model used some explicit and reasonable assumptions (discussed in the section on Sensitivity analysis). As per estimating the future projections of diagnosed diabetes cases in Al-Ahsa for 2023-2045, the latest IDF projections for KSA were used for standardization. The IDF rates of increase in the number of cases (≥ 20 years old) in KSA between 2022-2030 and 2030-2045 [1] were calculated and applied to estimate the number of cases in Al-Ahsa for the same years assuming a linear increase. It is important to note that each year in the model represents an independent modeling cycle, assuming that the mortality of diabetics was taken into account each year by IDF estimates, which were used to standardize the annual number of diabetes cases in Al-Ahsa. Table 1 and Table 2 summarize the rates of increase in diabetes cases during 2022-2045 as estimated by IDF and transition parameters used in the model.

**Table 1:** Summary of rates of the increase in the number of diabetic cases

Period	Rate of increase in the number of diabetic cases (%)	Reference
2022-2030	131.7	[1]
2030-2045	133.9	[1]
* Number of cases for each year between 2022 and 2045 was estimated using linear interpolation.		

**Table 2:** Summary of transition parameters used in the model

Parameter	Value	Used to inform the transition from : to	Reference
Incidence rate of major amputations in patients with diabetes (per 100,000 patients)	94.82	Diabetes : Major Amputations	[18]
Incidence rate of minor amputations in patients with diabetes (per 100,000 patients)	139.97	Diabetes : Minor Amputations	[18]
All- cause mortality rate in diabetic patients with amputations (per 1,000 person-years)	86.8	Major Amputations : Deaths in diabetics with amputations; Minor Amputations : Deaths in diabetics with amputations	[17]
All- cause mortality rate in diabetic patients without amputations (per 1,000 person-years)	23.81	No Amputations : Deaths in diabetics without amputations	[17]

Sensitivity analysis

Two methods of sensitivity analysis were conducted in order to estimate the uncertainty intervals (UIs) around the model outputs. The first method is 'analysis of extremes', where all model parameters were allowed to fluctuate 20% higher and 20% lower than the base-case outputs with keeping the original incidence rates constant over the whole modeling period. The second method is 'Scenario analysis', where the incidence rates of both major and minor amputations among diabetics were assumed to show a decline of 20% every three years of the modeling period. This assumption was based on some published evidence showing that, in most countries, where data are available, the incidence of DRA seems to be decreasing [4]. Unfortunately, there are no prospective studies measuring the DRA incidence in KSA or other countries in the MENA region. However, studies from other countries in Asia, Europe, and North America showed that incidence rates of major and minor amputations decreased by 12.3-28.1% and around 14%, respectively, when estimated over a three-year period duration [19-21]. Thus, the model was run using this scenario, where outputs with their UIs were estimated accordingly.

Model validation

The model was validated by comparing its main outputs with the actual 'observed' local data. The total number of DRA in Al-Ahsa was obtained from the two local governmental hospitals (King Fahad Hospital and Prince Saud Bin Jalawi Hospital), in which diabetic patients eligible for DRA are operated. The total number of amputations for 2022 was matched with that estimated by the model for the same year. Unfortunately, detailed data for categorizing amputations into major and minor in the two hospitals were incomplete, so a comparison was made for the total number only.

## Results

The total number of diagnosed diabetes cases (aged ≥20 years) in Al-Ahsa for 2022 was 54,805, as obtained from the Community Wellness Department, Al-Ahsa Health Cluster. This number is predicted by the model to increase to 72,178 in 2030 and 96,647 in 2045 assuming the same rates of linear increase by IDF. The annual projected number of DRA and mortalities varies according to whether the base-case scenario was used (the original incidence rates of major and minor amputations are constant), or the alternative scenario of decreasing these incidence rates by 20% every three years was applied.

Data in Table 3 show that, assuming constant baseline original incidence rates from 2022 to 2045, the model projected that the total number of DRA cases will increase from 129 (UI: 103-154) in 2022 to 169 (UI: 136-203) in 2030 and 227 (UI: 182-272) in 2045. Among these total cases, major amputation cases are estimated at 52 (UI: 42-62) in 2022, 68 (UI: 55-82) in 2030, and 92 (73-110) in 2045. On the other hand, minor amputations will increase from 77 (UI: 61-92) in 2022 to 101 (UI: 81-121) in 2030 and 135 (UI: 108-162) by 2045. The number of all-cause mortalities among amputees is predicted to increase from 11 (UI: 9-13) in 2022 to 20 (UI: 16-24) in 2045. Moreover, the number of deaths among non-amputees is forecasted to rise from 1,302 (UI: 1041-1562) in 2022 to 2,296 (UI: 1837-2755) by 2045.

**Table 3:** Summary of the main model outputs based on assuming constant incidence rates of major and minor amputations

Year	Total number of diabetes cases (n)	Total number of DRA (n, UI)	Total number of major DRA (n, UI)	Total number of minor DRA (n, UI)	All-cause mortality in amputees (n, UI)	All-cause mortality in non-amputees (n, UI)
2022	54,805	129 (103–154)	52 (42–62)	77 (61–92)	11 (9–13)	1302 (1041–1562)
2026	63,492	149 (119–179)	60 (48–72)	89 (71–107)	13 (10–16)	1508 (1207–1810)
2030	72,178	169 (136–203)	68 (55–82)	101 (81–121)	15 (12–18)	1715 (1372–2057)
2034	78,703	185 (148–222)	75 (60–90)	110 (88–132)	16 (13–19)	1870 (1496–2243)
2038	85,228	200 (160–240)	81 (65–97)	119 (95–143)	17 (14–21)	2025 (1620–2429)
2042	91,753	215 (172–259)	87 (70–104)	128 (103–154)	19 (15–22)	2180 (1744–2615)
2045	96,647	227 (182–272)	92 (73–110)	135 (108–162)	20 (16–24)	2296 (1837–2755)

Table 4 summarizes the main model outputs based on assuming declines in incidence rates of major and minor amputations by 20% every three years during 2022-2045. It is predicted that the number of DRA cases will drop from 103 (UI: 82-124) in 2022 to 91 (UI: 73-110) in 2030 and 61 (UI: 49-74) in 2045. The number of major amputations will decrease from 42 (UI: 33-50) to 30 (UI: 24-36) in 2030 and 13 (UI: 11-16) in 2045. Minor amputation cases are estimated at 61 (UI: 49-74) and 48 (UI: 39-58) by 2045. The number of deaths among patients with DRA is projected to be nine (UI: 7-11) in 2022 and five (UI: 4-6) in 2045, while deaths among non-amputees will increase from 1,302 (UI: 1,042-1,563) in 2022 to 2,300 (UI: 1,840-2,760) in 2045.

**Table 4:** Summary of the main model outputs based on assuming declines in incidence rates of major and minor amputations by 20% every three years

Year	Total number of diabetes cases (n)	Total number of DRA (n, UI)	Total number of major DRA (n, UI)	Total number of minor DRA (n, UI)	All-cause mortality in amputees (n, UI)	All-cause mortality in non-amputees (n, UI)
2022	54,805	103 (82–124)	42 (33–50)	61 (49–74)	9 (7–11)	1302 (1042–1563)
2026	63,492	98 (78–117)	36 (29–43)	62 (49–74)	8 (7–10)	1509 (1208–1811)
2030	72,178	91 (73–110)	30 (24–36)	61 (49–73)	8 (6–10)	1716 (1373–2060)
2034	78,703	82 (66–99)	24 (20–29)	58 (46–69)	7 (6–9)	1872 (1498–2246)
2038	85,228	74 (59–89)	20 (16–24)	54 (44–65)	6 (5–8)	2028 (1622–2433)
2042	91,753	67 (53–80)	16 (13–19)	51 (41–61)	6 (5–7)	2183 (1746–2620)
2045	96,647	61 (49–74)	13 (11–16)	48 (39–58)	5 (4–6)	2300 (1840–2760)

**Figure 2:**
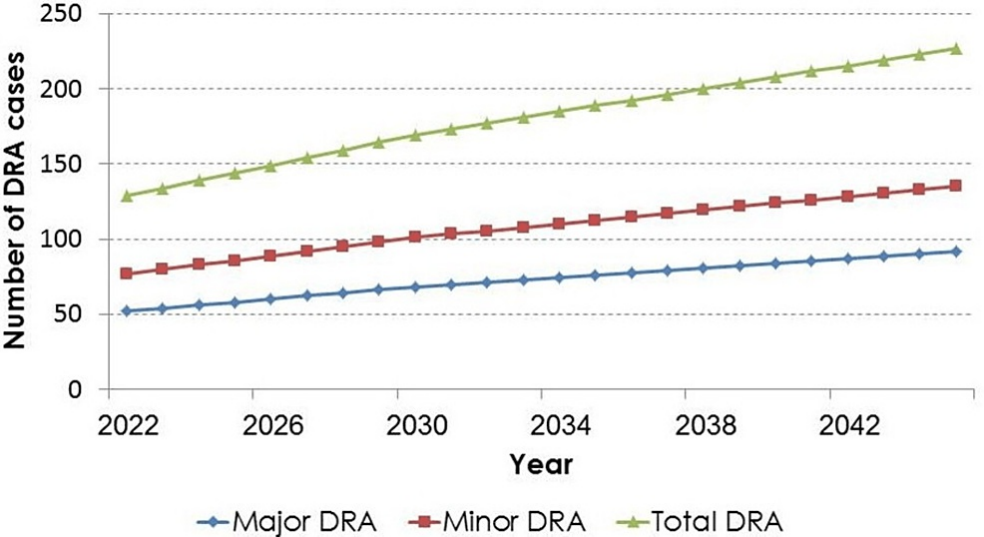
Future trends in the number of DRA cases assuming constant incidence rates

**Figure 3:**
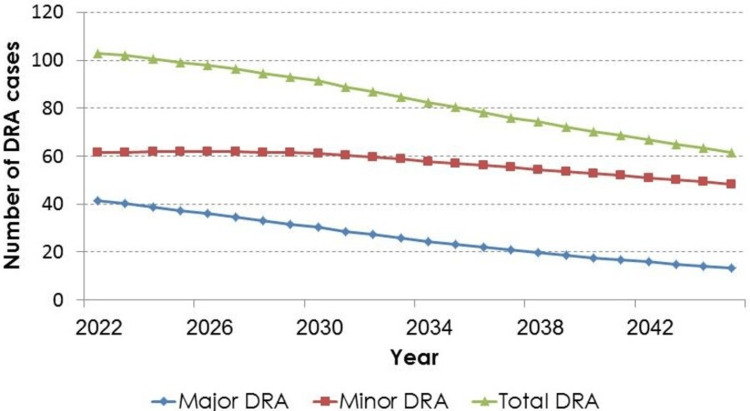
Future trends in the number of DRA cases assuming declines in incidence rates by 20% every three years

Figure 2 and Figure 3 present the graphical illustrations of the trends in the number of DRA cases (major, minor, and total) from 2022 to 2045, assuming the previously mentioned base-case original scenario and alternative scenario, respectively.

**Figure 4:**
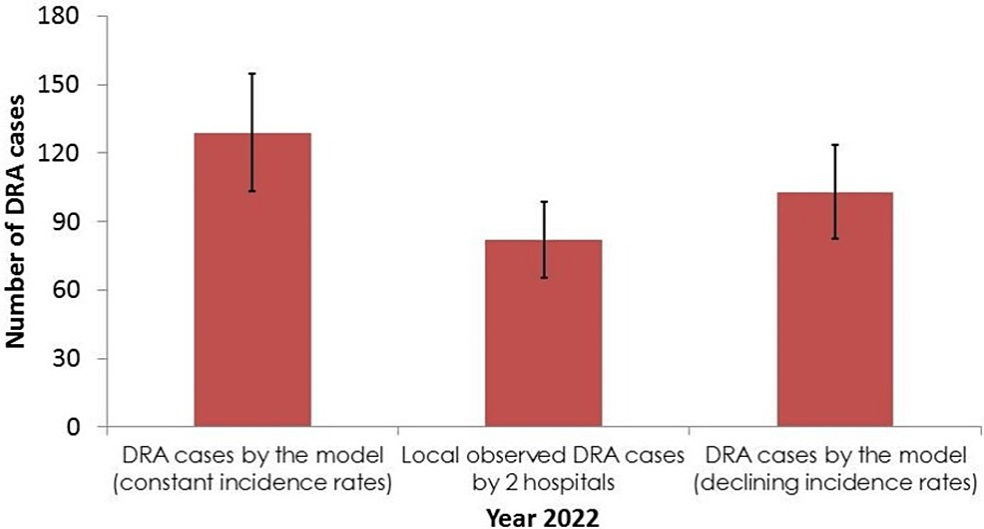
Comparison of the number of DRA cases between locally observed data and the model projections for 2022

For the purpose of validating the model, Figure 4 presents a comparison of the total number of DRA cases for 2022 as projected by the model with the observed data from the main two hospitals performing DRA in Al-Ahsa. There were a total of 82 DRA cases reported by the two hospitals in 2022 (73 cases from King Fahad Hospital and nine cases from Prince Saud Bin Jalawi Hospital). The model projected number of cases is 129 (47 more cases) if constant incidence rates are assumed. However, assuming the gradual decrease in incidence rates, the projected number is 103 (21 more cases) with the observed data lying within the UI of the model estimate.

## Discussion

In this study, a new epidemiological simulation model was developed and validated to provide future estimates of the burden of DRA in Al-Ahsa, Eastern Saudi Arabia. The model used two plausibly assumed scenarios for predictions, where the original incidence rates of major and minor amputations among diabetics, as obtained from recent evidence, are assumed to be either constant or gradually decreasing during the modeling period. The model also utilized published data to estimate the projections in the number of diabetes cases and to inform the transitions to death states. Between 2022 and 2045, the number of DRA cases among adults aged ≥20 years in Al-Ahsa is predicted to increase by around 175% (from 129 to 227) assuming unchanged incidence rates. However, this number is estimated to fall by approximately 40% (from 103 to 62) assuming the other scenario.

The model appears to produce higher estimates of the total number of DRA cases in Al-Ahsa when compared to the actual data from the two main hospitals, using the original constant incidence rates. Three possible explanations can be considered for this finding. First, it could reflect a true overestimate, which might be caused by overestimated incidence rates from the literature [18], as national rates from KSA or the MENA region are lacking. Second, the future projections for the number of diabetes cases may have inflated the true future size of diabetic individuals in Al-Ahsa. However, this number is projected using the same annual rates of increase generated by IDF [1], which are actually criticized by some studies for underestimating the true prevalence of diabetes in KSA [22]. Third, in addition to the two main governmental hospitals, there are four private hospitals in which DRA surgeries are carried out. Therefore, the observed cases of DRA in this study for 2022 are certainly underestimates of the actual situation. Data from these four private hospitals were not included for comparison in this study because of difficulties in accessing their records. However, it is very unlikely that the number of DRA cases in these hospitals will yield a substantial increase to the current observed data, because such surgeries are costly and, hence, the majority of operated patients in these hospitals should possess private medical insurance to cover the costs.

The alternative scenario of a gradual decrease in the original incidence rates seems to be more plausible when comparing the model outputs with the observed data. This conclusion is mainly supported by two considerations. First, as mentioned earlier, evidence from different regions of the world shows such a gradual decline in incidence rates over the past few years, particularly in high-income countries [4,19-21,23,24]. This is mainly attributed to improving healthcare for diabetic patients, which includes multidisciplinary diabetes clinics offering health education and foot care services [25]. A study from England has found that the three‐year diabetes‐related major amputation incidence correlated inversely with adequate delivery of diabetes foot care services [26]. Second, the model resulted in 21 more DRA cases for 2022 than those reported by the two governmental hospitals. This excess number in projected cases by the model does not appear to be substantial and is most likely achievable by adding the DRA cases from the other four private hospitals.

There is only one study from Saudi Arabia [27] that can be used for comparison with results from this model. In that study, the likely annual prevalence rates of DRA in Jeddah, Riyadh, and KSA were estimated by analyzing secondary data from a local database, published literature, and the annual reports of the Saudi Ministry of Health. The estimated annual prevalence rates of DRA (per 10,000 population aged ≥20 years) were 1.56, 2.35, and 2.6 in Jeddah, Riyadh, and KSA, respectively [27]. In our study, the estimated prevalence of DRA in Al-Ahsa (per 10,000 population aged ≥20 years as obtained from the latest national census) [28] for the first year of the modeling period (2022) is 1.82 assuming constant incidence rates and 1.45 assuming a gradual decrease in incidence rates. Hence, the model in this study appears to produce an almost consistent prevalence of DRA in Al-Ahsa as that previously estimated for Jeddah and slightly lower than that reported for Riyadh and overall KSA.

It is difficult to validate the number of mortalities predicted by the model because, obviously, there is a lack of reliable data on specific causes of death reported in death certificates in most countries globally [29,30]. However, all-cause mortalities in this study provide a useful primary measure of the burden of DRA and were estimated by applying national rates obtained from a relatively recent study using a nationwide sample of diabetics in KSA [17].

To set proper preventive services and allocation of resources, this study offers the policymakers a new useful resource for forecasting the future burden of DRA in Al-Ahsa. However, some limitations should be noted. First, the model used a simplistic and crude method of future projections, and, as in all modeling studies, outputs are conditional upon the data inputs and assumptions used. However, as discussed earlier, it has been attempted to obtain data from recent credible sources and to make explicit and justifiable assumptions. Second, various data sources might be associated with varying data quality. Again, this is inherent to all modeling studies and was managed by sensitivity analysis to determine the potential uncertainties around the model outputs. Third, the burden of DRA was not stratified by sex or age groups because of insufficient relevant data from the utilized literature.

Several recommendations can be proposed based on the findings of this study. First, further research studies are required to construct more complex models that incorporate risk factors of DRA in the modeling process, stratify the data inputs by sex and age groups, or apply a life-table-based approach. Future research studies can also focus on identifying the levels of specific risk factors affecting the incidence of DRA, such as diabetes management, access to healthcare, and socioeconomic and lifestyle factors. Second, preventive measures to tackle the impact of diabetes complications, including DRA, are urgently needed. These measures may include public awareness campaigns about diabetes prevention, early detection and management of diabetes, promoting healthy lifestyles, and improving access to diabetes education and healthcare services. Third, some policy actions that support diabetes prevention and management should be empowered. This may include increasing funding for diabetes research, improving access to diabetes medications and devices, implementing regulations to promote healthy food environments, and supporting initiatives to address social determinants of health.

## Conclusions

DRA impose a considerable burden on patients and the healthcare system, even with the possibility of a potential decrease in incidence rates. Multi-sectorial efforts toward preventive and curative healthcare services offered to individuals with diabetes in Saudi Arabia should be intensified in order to minimize the burden of diabetes and its complications including DRA.
